# Detection of *Cercopithifilaria bainae* infection in shelter dogs and ticks in Oklahoma, USA

**DOI:** 10.1186/s13071-020-04089-z

**Published:** 2020-04-25

**Authors:** Megan W. Lineberry, Kellee D. Sundstrom, Susan E. Little, Erin M. Stayton, Kelly E. Allen

**Affiliations:** Department of Veterinary Pathobiology, Oklahoma State University College of Veterinary Medicine, Stillwater, OK USA

**Keywords:** *Cercopithifilaria bainae*, Dermal punch biopsy, Filarioid, Microfilariae, Mitochondrial *12S* rDNA, *Rhipicephalus sanguineus* sensu lato, Saline sedimentation, Third-stage larvae

## Abstract

**Background:**

*Cercopithifilaria bainae* is a filarioid nematode of dogs. Infection with the parasite was not reported in the USA until 2017, when a dog with skin lesions in Florida was diagnosed. Brown dog ticks, *Rhipicephalus sanguineus* (*sensu lato*), are the purported tick vectors, and are widespread in the USA. Therefore, *C. bainae* is likely present in additional states. Here, we tested dogs and ticks in Oklahoma for evidence of *C. bainae* infection.

**Methods:**

Dermal punch biopsies were opportunistically collected from municipal shelter and client-owned dogs. Multiple skin samples collected from interscapular and head regions were tested by saline sedimentation to recover live microfilariae for morphometric identification and by PCR to amplify a 330 bp region of the filarioid *12S* rRNA gene. Also, ticks observed on surveyed dogs were collected, identified to species level, and tested for filarioid DNA.

**Results:**

A total of 496 saline sedimentations were performed on 230 shelter and 20 client-owned dogs. *Cercopithifilaria bainae* infections were identified in 2.6% (6/230) of shelter dogs by morphometry of microfilariae in sedimentations and/or amplification of DNA from skin. DNA sequences amplified from PCR positive skin samples were 99–100% identical to *C. bainae* reported in Italy. All skin samples from client-owned dogs were negative for filarioid infection by saline sedimentation and PCR. A total of 112 ticks, comprised of four species, were collected. Two of 72 *R. sanguineus* (*s.l*.), both engorged females found attached to a *C. bainae* infected dog, harbored *C. bainae* DNA (99–100% identity). One attached *R. sanguineus* (*s.l*.) male on the same dog harbored filarioid DNA sequence which was difficult to interpret at numerous base-pair locations, but was closest in identity (~80%) to *C. bainae*.

**Conclusions:**

The distribution of *C. bainae* is more widespread than previously known. To our knowledge, we document *C. bainae* infections in dogs and DNA in brown dog ticks in Oklahoma for the first time. As brown dog ticks are commonly found throughout the USA, veterinarians in this region should consider *C. bainae* infection as a differential diagnosis in canine patients with dermatitis or polyarthritis.
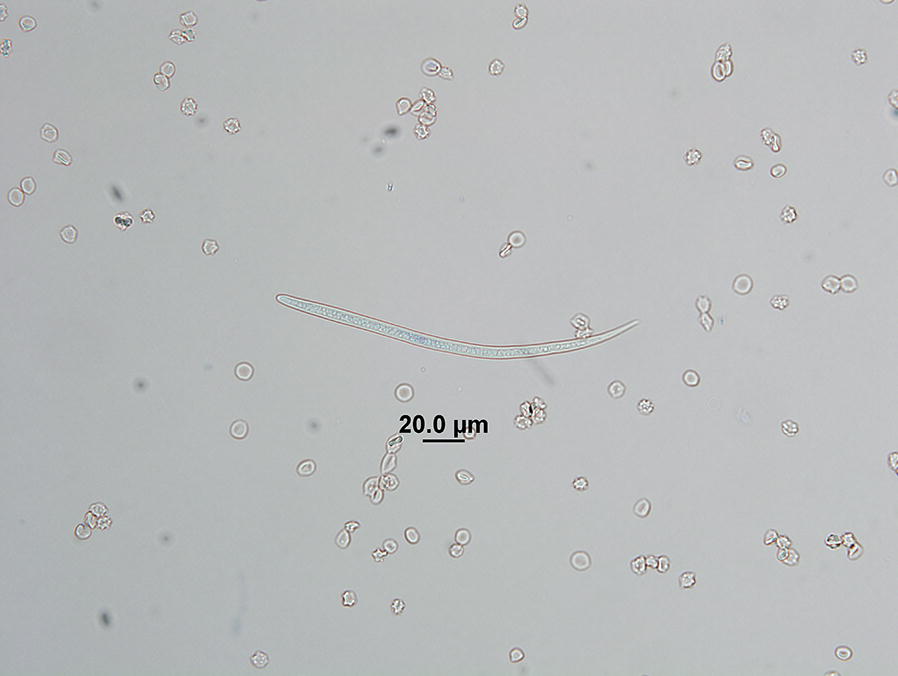

## Background

*Cercopithifilaria bainae* is a tick-borne filarial nematode of dogs that was first described in Brazil in 1984 [1]. Adults of *C. bainae* parasitize the subcutaneous tissue of canine hosts, and microfilariae remain sequestered in the dermis, making detection of the parasite in infected dogs challenging [2]. *Cercopithifilaria bainae* is considered primarily non-pathogenic, but erythematous, papular and pruritic dermatitis, non-healing and ulcerative skin lesions, and subcutaneous nodules associated with infection have been reported [3–5]. One case of polyarthritis has also been documented [3].

*Cercopithifilaria bainae* infections in dogs have been documented predominantly in Mediterranean Europe and in Brazil, and DNA of the parasite has been reported in the suspected tick vector, *Rhipicephalus sanguineus* (*sensu lato*), collected in these areas. DNA of *C. bainae* has also been identified in *R. sanguineus* (*s.l*.) collected in other regions, including Australia, Malaysia and South Africa [3, 4, 6, 7]. *Rhipicephalus sanguineus* (*s.l*.), commonly called brown dog ticks, are thought to be important natural vectors of *C. bainae* based on the development of third-stage larvae in adult ticks acquisition fed as nymphs on a naturally infected dog [8]. Although *C. bainae* has been molecularly detected in other ticks, including *Dermacentor reticulatus* and *Ixodes ricinus*, parasite development within these tick species has not been experimentally demonstrated [8, 9].

Despite the cosmopolitan distribution of brown dog ticks, *C. bainae* had not been documented in the USA until 2017. A dog from Florida with no travel history was presented with dermatitis, with plaques on the dorsal head, and alopecia, erythema, and ulceration of both medial canthi. Microfilariae isolated from skin biopsy samples via saline sedimentation were identified as *C. bainae* by PCR and microscopy [5].

Brown dog ticks are widespread in the USA, with all stages preferentially feeding on dogs, and it is likely that *C. bainae* is present in dogs in states in addition to Florida [10, 11]. To the authors’ knowledge, however, no studies investigating geographic distribution of *C. bainae* in dogs in the USA have been conducted. Raising awareness of the emerging parasite in the USA will assist veterinarians in diagnosing infections, which will generate further information regarding clinical manifestations and pathology in infected dogs, and lead to investigations into treatment and prevention strategies. To determine if *C. bainae* is present in dogs in Oklahoma, multiple dermal punch biopsy samples were evaluated by saline sedimentation and PCR. Additionally, ticks observed on dogs were tested for filarioid DNA.

## Methods

### Skin biopsy sample collection

Skin biopsy samples were opportunistically collected from euthanatized dogs in Oklahoma, USA, over a 10-month period (January-October 2018). Shelter dogs were temporarily housed at animal control facilities prior to euthanasia following standard approved shelter protocols and client-owned dogs were submitted for necropsy at the Oklahoma Animal Disease Diagnostic Laboratory (Payne County, Stillwater, OK). When possible, sex and estimated age were documented. Travel histories were not available for the majority of animals, nor was information regarding prior treatment with parasiticides.

Multiple skin samples were collected from individual animals using sterile 6 mm biopsy punches within hours, but sometimes up to four days, after death. Carcasses were stored at 4 °C until sample collection. Higher frequency of *C. bainae* microfilariae in interscapular and head regions has been previously described [12], and therefore these focal regions were sampled; up to four interscapular and up to three head samples were collected from each animal. At times of skin biopsy sample collection, the skin was briefly examined for cutaneous nodules and other lesions.

Single interscapular biopsy samples were placed in microcentrifuge tubes containing phosphate-buffered saline (PBS) and transported to the laboratory for storage at − 20 °C and later DNA extraction and molecular analyses. Additional biopsy samples were placed in PBS-filled, sterile 15 ml conical tubes and, upon transport to the laboratory, processed to recover microfilariae as described below. After processing, the majority of skin samples were stored at − 20 °C for subsequent DNA extraction and PCR.

### Saline sedimentation of skin biopsy samples

To detect microfilariae in skin biopsy samples, up to three skin samples from individual dogs were placed in 15 ml conical tubes containing PBS and incubated for 1–3 h at 37 °C to allow live microfilariae to migrate out of the tissue [12]. The skin was removed, and remaining PBS was centrifuged at 388× *g* for 5 min to concentrate microfilariae. Supernatants were decanted and resulting pellets were stained with 0.1% methylene blue for microscopic examination.

Stained sediment was transferred to microscope slides and covered with 22 × 60 mm glass coverslips; all sedimentation material from each skin sample was scanned under 100× total magnification. When observed, microfilariae on slides were enumerated, and up to 10 microfilariae were measured using an ocular micrometer (length and width) under 400× total magnification. Microfilariae measurements were compared to those available in the literature identifying filarioid species including *Acanthocheilonema reconditum* (215–288 × 4.5–5.8 µm), *Cercopithifilaria bainae* (173.8–200 × 5.6–6.9 µm), and *Dirofilaria immitis* (280–325 × 5–7.5 µm) [13–15]. Microfilariae in sedimentations were gently washed from slides with PBS and stored at 4 °C for DNA extraction within 48 h for subsequent molecular identification.

### Tick collection and processing

Animals were briefly examined (approximately 1–3 min) for ticks at the time of skin biopsy collection. When present, ticks were placed in 70% ethanol and stored at − 20 °C. At the time of dissection, ticks were removed from ethanol and identified to species by microscopic examination and comparison with standard keys [16]. Identified ticks were then individually dissected and internal contents removed and digested in Proteinase K and lysis buffer solution at ambient temperature [17].

### DNA extraction methods, PCR, and sequence analysis

Tick dissection, DNA extraction, PCR amplification, and amplicon purification were carried out in dedicated laboratory areas to prevent DNA contamination. Separate negative water controls were used for DNA extractions and for PCR. A sample containing DNA of *D. immitis* was used as a positive control.

Nucleic acid was extracted from approximately 30 mg sections of skin biopsy samples using the QIAamp® Fast DNA Tissue Kit (Qiagen, Valencia, CA, USA). Refrigerated microfilariae (washed with PBS from glass microscope slides) were extracted for DNA using the Illustra^TM^ blood genomicPrep Mini Spin Kit (GE Healthcare, Piscataway, NJ, USA). After tissue digestion, individual tick samples were extracted for DNA using the QIAamp® DNA Blood Mini Kit (Qiagen, Valencia, CA, USA). DNA extractions were carried out according to the manufacturer’s instructions specific to each kit.

PCR amplifying a ~ 330-bp region of the filarioid *12S* rRNA mitochondrial gene was performed on DNA extractions from skin, microfilariae, and ticks using previously described primers Fila12SF and Fila12SR [2]. Individual reactions were carried out in a total volume of 25 µl containing 1× AmpliTaq Gold 360 (Applied Biosystems, Carlsbad, CA), 0.8 µM of each primer, and 2 µl of DNA. Thermocycler conditions were as follows: 94 °C for 10 min, followed by 40 cycles of 94 °C for 45 s, 52 °C for 45 s, and 72 °C for 90 s, and ending with a final extension step of 72 °C for 7 min.

Additionally, PCR amplifying a 340–370-bp region of the *12S* rRNA mitochondrial gene was performed on *R. sanguineus* (*s.l*.) testing positive for *C. bainae,* using previously described primers 12SF and 12SR, to determine the genetic lineage (temperate or tropical) of the ticks as previously described [17, 18].

Standard gel electrophoresis in a 2% agarose matrix with GelRed® staining (Biotium, Fremont, CA) was used to detect amplicons. Correctly sized amplicons were purified either directly from the gel using the QIAquick® Gel Extraction Kit (Qiagen) or from PCR reactions using the QIAquick® PCR Purification Kit (Qiagen).

Purified amplicons were bi-directionally sequenced (Sanger method) by Eurofins Genomics (Louisville, KY) or the Oklahoma State University Molecular Core Facility (Stillwater, OK). Sequences from skin samples and ticks were compared to those available in the National Center for Biotechnology Information database (GenBank^TM^) to determine filarioid species identity and *R. sanguineus* (*s.l*.) genetic lineage. Sequence alignments were constructed using ClustalW to determine percent similarities of Oklahoma filarioid *12S* rRNA mitochondrial gene sequences to each other and to additional filarioid sequences previously contributed to the GenBank^TM^ repository, as well as to determine *R. sanguineus* (*s.l*.) genetic lineage.

## Results

### Dogs surveyed

The sample set included 230 shelter dogs and 20 owned dogs. Shelter dogs consisted of 55.2% (127/230) males and 43.5% (100/230) females, with reported ages ranging from two months to 14 years ($\bar{x}$ = 2.4 years; 95% CI: 2.00–2.87). Sex was not recorded for three dogs and age was not reported for 106 dogs. One of the shelter animals was a coyote, but was included as part of the shelter cohort. Four shelter dogs tested positive for circulating heartworm antigen on a commercial patient-side diagnostic assay conducted by the shelter. A single shelter dog was noted to have alopecia and scabbing on the face and dorsum at the time of skin biopsy collection. Although sometimes difficult to ascertain in some animals due to overall poor condition, cutaneous lesions were not noted on other dogs. Owned dogs consisted of 10 males and 10 females, with reported ages ranging from 3 months to 12 years ($\bar{x}$ = 6.4 years; 95% CI: 4.14–8.73). Age was not reported in one dog. Necropsy revealed that two owned dogs were infected with adult *D. immitis*. No dermatological lesions were reported for any of the owned animals.

### Microfilariae recovered in saline sedimentations

A total of 496 saline sedimentations were performed on 230 shelter dogs and 20 owned dogs. Microfilariae were recovered from 8.7% (20/230) of shelter dogs. A total of eight microfilariae recovered from 1.3% (3/230) of dogs were consistent with *C. bainae* by morphometry, measuring 173–200 × 5.6–7.5 µm. Body regions where *C. bainae* microfilariae were recovered and number of microfilariae recovered in individual biopsy samples are included in Table 1. *Demodex* sp. was recovered by saline sedimentation from the single shelter dog with obvious skin lesions (alopecia and scabbing). No microfilariae were recovered from skin biopsy samples collected from client-owned dogs.

**Table 1:** *Cercopithifilaria bainae* infections in dogs in Oklahoma, USA

Dog ID	Head region		Interscapular region	
	Microfilaria	PCR	Microfilaria	PCR
60	−	−	−	+
85	1	−	−	−
88	−	−	−	+
105	−	+	−	−
112	1	−	4	+
220	−	−	2	+

*Acanthocheilonema reconditum* (215–288 × 4.5–5.8 µm) was identified in 1.3% (3/230) of dogs and *D. immitis* (280–325 × 5.0–7.5 µm) was identified in 5.2% (12/230) of dogs. One dog had a single microfilaria recovered from the interscapular region that desiccated on the slide, so an accurate measurement was not possible for species determination. This dog was later confirmed as having *D. immitis* by PCR of the sediment washed from the slide with PBS. One microfilaria (measuring 160 × 4.5 µm) recovered from a single shelter dog did not fall into known filarioid microfilariae size ranges.

On average, the numbers of microfilariae detected for *A. reconditum* and *D. immitis* in individual skin biopsy samples were higher when compared to *C. bainae*, which ranged in number from one to four. Of the dogs with *A. reconditum* or *D. immitis*, 93.3% (14/15) had detectable microfilariae in interscapular regions, ranging in number from one to 68, and 80% (12/15) had detectable microfilariae in head regions, ranging in number from one to 239.

DNA from microscopically identified *C. bainae* or *A. reconditum* microfilariae was not detectable in material rinsed from sedimentation slides. DNA of *D. immitis* microfilariae rinsed from slides was detected in 55% (11/20) of samples.

### PCR of skin biopsy samples

Skin samples from 228 shelter dogs and eight owned dogs were tested by PCR, all of which were also tested by saline sedimentation. A total of 9.6% (22/228) of shelter dogs were positive for filarioid DNA, with 2.2% (5/228) having DNA of *C. bainae* (Table 1); two of these dogs were also positive for *C. bainae* microfilariae by microscopy. *Acanthocheilonema reconditum* and *D. immitis* DNA was also detected in 0.9% (2/228) and 6.6% (15/228) of dogs, respectively. When assessing dermal areas of skin biopsy collection, 8.3% (19/228) of dogs had detectable DNA in the interscapular region, including 1.8% (4/228) with *C. bainae*, 0.9% (2/228) with *A. reconditum,* and 5.7% (13/228) with *D. immitis* infections. In the head region, 4.8% (11/228) of dogs had detectable DNA, including 0.4% (1/228) with *C. bainae*, and 4.4% (10/228) with *D. immitis* infections. *Cercopithifilaria bainae* sequences obtained from shelter dogs were 99–100% homologous to each other and to *C. bainae* reported in Italy (GenBank: KF381408). *Cercopithifilaria bainae* sequences obtained from dogs in this study were submitted to GenBank^TM^ (MN814265-MN814269). *Acanthocheilonema reconditum* and *D. immitis* sequences were 99–100% homologous to GenBank^TM^ accessions JF461460 and MH051846, respectively. None of the samples from owned dogs had detectable filarioid DNA by PCR.

### PCR of dissected ticks

A total of 112 ticks were collected from 17 dogs, including two dogs with *C. bainae*. A total of 110 ticks were collected from 16 shelter dogs (16/230, 7.0%) and were comprised of *Amblyomma americanum* (1 nymph, 10 males, 6 unengorged females and 8 engorged females), *Amblyomma maculatum* (5 males and 1 unengorged female), *Dermacentor variabilis* (4 males, 1 unengorged female and 3 engorged females), and *R. sanguineus* (*s.l*.) (47 males, 3 unengorged females and 22 engorged females). Two partially engorged *A. americanum* females were collected from one (1/20, 5.0%) client-owned dog.

Two shelter dogs with *C. bainae* microfilariae by sedimentation were noted to have *R. sanguineus* (*s.l*.) on them at the time of skin biopsy sample collection; three attached, engorged females and two attached males were collected from one of these dogs. Two of the engorged *R. sanguineus* (*s.l*.) harbored DNA sequences that were 99% identical to each other and 99% homologous to *C. bainae* from Italy (KF381408); *C. bainae* sequences from the female *R. sanguineus* (*s.l*.) were 99–100% identical to *C. bainae* sequences amplified from skin of dogs in this study. One of the male *R. sanguineus* (*s.l*.) harbored sequence that was difficult to interpret at numerous base-pair locations due to heterozygous and mis-spaced peaks, suggesting co-infection with similar organisms, but was closest in identity (~80%) to *C. bainae* (GenBank: KF381408). Attempts to clone amplicons from the male *R. sanguineus* (*s.l*.) into plasmid vectors to better elucidate nucleotide sequences of single gene fragments were unsuccessful. The *R. sanguineus* (*s.l*.) ticks which harbored *Cercopithifilaria* sp. sequences were identified as belonging to the temperate lineage. The *R. sanguineus* (*s.l*.) ticks which tested negative for *Cercopithifilaria* sp. DNA were not tested for genetic lineage. Both *A. americanum* collected from the client-owned dog were negative for filarioid DNA by PCR.

In addition to detection of *Cercopithifilaria* sp. DNA in ticks, DNA of *D. immitis* was detected in 13 ticks collected from six different dogs, including five *A. americanum* (2 males, 1 unengorged female and 2 engorged females) and eight *R. sanguineus* (*s.l*.) (4 males and 4 partially to fully engorged females). Two dogs with detectable *D. immitis* DNA in infesting ticks were positive for *D. immitis* by skin sedimentation and/or PCR, two dogs did not have microfilariae or detectable filarioid DNA in skin, and two dogs with *D. immitis* positive ticks were positive for *C. bainae* microfilariae by skin sedimentations and/or PCR. No *A. reconditum* DNA was detected in any of the ticks tested.

## Discussion

*Cercopithifilaria bainae* infections in dogs have predominantly been identified in Mediterranean Europe and Brazil, with detection in 10.5–13.9% and 1% of dogs surveyed, respectively; these are regions where researchers have been actively looking for the parasite [7, 19]. However, due to the cosmopolitan distribution of *R. sanguineus* (*s.l*.), the experimentally demonstrated tick vector, it is logical to deduce that *C. bainae* infections in dogs are similarly distributed, as are other infections transmitted by this tick group including *Anaplasma platys*, *Ehrlichia canis*, canine *Babesia* spp. and *Hepatozoon canis* [10, 20–22]. Here, we report *C. bainae* in dogs in Oklahoma for the first time, only the second documentation of the parasite in North America. The parasite was detected in 2.6% (6/230) of shelter dogs when PCR and sedimentation results are considered together. Although PCR and sedimentation results in the present study did not always agree, discrepant results between PCR and sedimentation assays have been documented previously in *C. bainae-*infected dogs [12]. It is also possible that the length of time between euthanasia and sample collection could have affected recovery of live microfilariae; the number of microfilariae recovered were lower than previously documented [12]. Longer durations of time may have led to parasite mortality and failure to migrate out of tissue. This may explain why some dogs in the study were positive for *C. bainae* by PCR of skin, but not by microscopy.

It is not surprising that filarioid infections were detected less commonly in client-owned dogs than shelter dogs. Owned dogs often receive more frequent veterinary care relative to shelter dogs, and therefore are more likely to be treated with compounds effective against helminths or ectoparasites [23, 24]. However, if approximately equal numbers of skin samples from pet dogs were tested, filarioid infections may have been detected in more animals within the cohort. Not unexpectedly, we also detected *A. reconditum* and *D. immitis* infections in shelter dogs in this study; both parasites are well-documented in the USA [25, 26]. *Acanthocheilonema reconditum* infections were identified in 2.2% (5/230) of shelter dogs, and were more commonly detected by skin sedimentation of the head region or PCR of skin samples collected from the interscapular region. The prevalence of *A. reconditum* infection in dogs in Oklahoma has not been reported. *Dirofilaria immitis* infections were identified in 8.3% (19/230) of shelter dogs, and were more commonly detected by PCR of interscapular skin samples rather than detection of microfilariae by sedimentation. The overall heartworm prevalence observed in shelter dogs in this study was comparable to what has been previously reported in Oklahoma shelter dogs [27].

To the authors’ knowledge, the present study is the first report of *Cercopithifilaria* sp. DNA in ticks in the USA, and suggests *R. sanguineus* (*s.l*.) may serve as vector in this region, as has been reported in other areas of the world [19]. All three of the PCR positive *R. sanguineus* (*s.l*.) were attached to one dog that was later determined to have *C. bainae* microfilariae; the female ticks were engorged, but it was not apparent for how long the male tick had been attached or if a blood meal was taken. The presence of *R. sanguineus* (*s.l*.) on a dog infected with *C. bainae* is noteworthy, and compels the authors to suspect that the parasite is cycling between this tick group and dogs in the USA. If *C. bainae* microfilariae had been ingested by immature *R. sanguineus* (*s.l*.) stages, they may have gone on to develop into infective third-stage larvae within ticks during ecdysis, as has been experimentally demonstrated in this tick group in other areas of the world [19].

Alternatively, the *Cercopithifilaria* sp. DNA amplified from the three ticks may have occurred following incidental ingestion of dermal microfilariae from the infected dog on which they were found. This possibility is evidenced by the fact that DNA of *D. immitis* was detected in 20% (5/25) of the *A. americanum* and 11.1% (8/72) of the *R. sanguineus* (*s.l*.) tested. As *D. immitis* is adapted to mosquito intermediate hosts [13], it is extremely unlikely that developing larvae were present within ticks, but rather microfilariae were incidentally ingested in blood. Although previous studies have demonstrated molecular evidence of *C. bainae* in other tick species (*Dermacentor reticulatus* and *Ixodes ricinus*), *R. sanguineus* (*s.l*.) is the only tick group which has been experimentally demonstrated to host developing stages of the parasite [8, 9]. In this study, *C. bainae* was not detected in *A. americanum*, *A. maculatum* or *D. variabilis*. However, if more specimens of each of these tick species were tested, then *C. bainae* DNA may have been detected, especially if ticks had recently fed on infected dogs.

## Conclusions

*Cercopithifilaria bainae* infections in dogs in the USA are more widespread than previously thought. Here, we document infections in dogs and DNA of the parasite in engorged *R. sanguineus* (*s.l*.), the experimentally demonstrated tick vector, in Oklahoma for the first time. Due to the ubiquity of *R. sanguineus* (*s.l*.), practicing veterinarians should consider *C. bainae* infection as a differential etiology when diagnosing canine dermatitis and polyarthritis, especially for those animals with known histories of brown dog tick infestations.

## Data Availability

The data supporting the conclusions of this article are kept on file in the KA laboratory and are available upon request for review by *bona fide* researchers. *Cercopithifilaria bainae* sequences obtained from dogs in this study were submitted to the GenBank database under the accession numbers MN814265-MN814269.
